# Validity and reliability of the Arabic version of Activities of Daily Living (ADL)

**DOI:** 10.1186/1471-2318-9-11

**Published:** 2009-03-29

**Authors:** Ramzi Nasser, Jacqueline Doumit

**Affiliations:** 1 Center of Educational Development and Research, Qatar University, POBOX 2713, Doha, Qatar; 2 Faculty of Nursing and Health Sciences, Notre Dame University, POBOX 72, Zouk Mosbeh, Lebanon

## Abstract

**Background:**

The Activity of Daily Living (ADL) is an instrument that screens elderly respondents for physical functioning and assesses whether they are dependent or independent in their daily activities. This study demonstrates a translation procedure and obtains the reliability and validity of a translated, Arabic ADL.

**Methods:**

The ADL was translated to Arabic through a forward translation method followed by a committee-consensual approach. The ADL and the Arabic Mini-Mental State Examination (AMMSE) were administered to an opportunistic sample of 354 Lebanese elderly living in nursing homes who did not have dementia.

**Results:**

Reliability split half measures, sensitivity, and negative predictive values were high across all dimensions of the ADL with the exception of feeding. There were non-significant differences on the scored ADL between the three age groups: young age, middle age and older old. In addition, a non-significant difference was found on the scored ADL between the high and low AMMSE scores.

**Conclusion:**

Overall, the translated ADL was consistent and valid measure for assessing daily activities in elderly nursing home residents. As it is quick and easy to use, the ADL in Arabic could help caregivers and doctors to prescribe appropriate physical exercise for elderly Arabic speaking patients.

## Background

In 2006, the number of people aged 60 years and above was 11.1% of the Lebanese population [1]. According to the World Health Organization [2] by 2025 there will be more than 800 million people worldwide over 65 years, two-thirds of whom will live in nations with developing economies. Plouffe [3] calculated that by 2030 the number of old persons in developing countries will be nine times greater than at present. Many of the elderly will demand better quality of life through palliative care, drugs, preventative medicine, and/or physical exercise [4] to help reverse impairment related to pathophysiological conditions [5].

Physical support facilities for elderly dependents are found in nursing homes (NHs) [6]. Generally, physicians and other clinicians involved in their care, such as dieticians, need rapid appraisal tools for diagnosing the elderly and assessing the effects of interventions. While geriatricians often screen the elderly for a particular health condition or a disease, they rarely rely on validated scales or self-rated assessments [7]. Perhaps the most difficult part of using subjective self-assessment tools in the elderly is the potential for fluctuation in their responses. Designing and adapting unambiguous items to tap into patient perspectives can help screen for conditions as in continence, not easily observable by clinicians.

Assessment by caregivers of the activities of daily living is important in identifying the degree of independence or dependence in NH residents [8]. The ADL instrument is a short assessment of functional ability or physical functioning for older persons [9]. Examples of similar scales include the Barthel Index [10], Kenny Self-Care Evaluation [11], Functional Status Index [12], and Functional Activities Questionnaire [13]. The advantage of the ADL [9] is that the elderly can be evaluated via the clinical questions over a full range of functional abilities.

The ADL scale was translated from English to Arabic then sampled among Arabic speaking elderly living in Lebanese NHs (See Additional file 1: Appendix). Several translation methods are available and the choice depends on the nature of the material being translated. One sound method is the convergent and discriminant validity paradigm, called the method of convergence [14] or panel design [15]. It involves having at least three judges rate the translations; if the judges agree that the translated items reflect the intentions of the originals, their judgments converge or are congruent (inter-subjective agreement) and provide an evidence for the logical validity of the translation. This basic model is logical and practical and has wider implications for validity.

In this study, the ADL was translated to Arabic and rated by judges on three translation factors: appropriateness, application, and adequateness. Next, the Arabic instrument was administered to elderly NH patients to obtain reliability and validity measures.

## Methods

The ADL assesses overall functional activity in 1) bathing, 2) dressing, 3) going to toilet, 4) transferring (movement), 5) continence, and 6) feeding. It was forward translated by the author, co-author, and a third academic. All translators were native speakers of Arabic and teach in English. Each of the three translators was blinded to the results of the other translators' efforts. To validate the Arabic translations, three judges rate each of the three translations. All judges were native speakers of Arabic and were proficient in Arabic and English. One judge had a PhD in biochemistry, the second was an MD specializing in geriatric medicine, and the third was a PhD in nutrition. All judges had more than 5 years experience working in academia, the geriatric and nutritionist, on a part-time basis, worked in NHs.

The judges, blinded to the translators and to each other's ratings, they rated the translated items for three factors: applicability (can these items be used by researchers in Lebanese NHs?), adaptability (are caregivers or the elderly able to answer or perform a task described in the items?), and suitability (are the items appropriate for the elderly in Lebanon?. Their ratings were then assessed on a three-point scale: high agreement between the raters, neutral, or low agreement. If all the judges agreed that a translation was applicable, adaptable, and suitable, it was used in a process of consensus making. If there was disagreement on whether a translated word or phrase on one of the ADL items was applicable, adaptable, or suitable, the judges were told that a disagreement was present and required to agree on a new translation by the three judges in a process of consensus making. A reiterative approach had the judges rating the items and changing the translations until full agreement between them was reached. The Arabic ADL was not back-translated but used in its final form in the Arabic language.

The total ADL score lies on an ordinal scale ascending from 0 to 6, where 6 entails complete independence and 0 complete dependence. The six individual components of the overall score are ordered, beginning with a lack of bathing ability, then dressing, going to the toilet, movement, continence, and, finally, feeding. The response format of the English ADL for each item was binary, either a 0 (complete dependence) or a 1 (complete independence). The responses on the Arabic version of the ADL were altered to 0, 0.5, or 1, with 0.5 indicating partial independence.

Functional impairment scores were calculated for each item and for the overall total. A total mean percentage score (hereafter referred to as the total score) was obtained by adding the scores on the 6 items, dividing by 6 and then multiplying by 100. The lower the total ADL score, the more severe the dependence. Using an adaptation of the model developed by Johnson et al., [16], a score of 0–33 indicated "severe dependence", 34–66 indicated "moderate dependence", and a score of 66 or above indicate "no to mild dependence".

Sensitivity and specificity are measures of accuracy: sensitivity refers to the proportion of patients with a given disease who have a positive test and specificity to the proportion of patients without the disease who have a negative test. Predictive value refers to the likelihood that a patient has or does not have the condition, given a positive or negative test result [17]. In analyzing each item, the 0.5 was transformed to a 0 when calculating sensitivity, specificity, positive predictive value and negative predictive value, such that partially dependent respondents screened positive and there was an increased risk of false positive results.

When testing the validity of the Arabic ADL, it was expected that a high sensitivity would indicate that elderly respondents who were dependent would screen positive and that there would be a low false negative rate [17]. To substantiate sensitivity and specificity, positive and negative predictive values were calculated. The sensitivity, specificity, and predictive values of the total ADL and its subscales were analyzed for two subgroups of respondents who had physical debilitating conditions, namely, being crippled (unable to walk either by from birth or subsequent injury) or having had an amputation (removal of body parts such as fingers, arms, or feet because of injury or disease). Respondents who were crippled or who had had an amputation were identified from the diagnoses made by a geriatrician and/or through direct observation.

### Sample and Ethical Considerations

A research grant review committee of the World Health Organization, Eastern Mediterranean Regional Office, granted approval to carry out the study. The study was supported by the Ministry of Social Affairs, Ministry of Public Health, and National Association of Elderly Affairs. The study was partially funded by the WHO through contract # EM/07/0564956. An ethics committee did not exist at the national level or where the two authors work.

In 2007 there were 44 NHs in Lebanon that assisted the elderly with feeding, shelter, physical support, and medical support. Seven field researchers moved from one region to another, starting in the North then going to the South, East, central Lebanon, and the capital, Beirut; and 36 NHs were visited.

Residents who agreed to be part of the study and did not fit five exclusion criteria were included in the study. The exclusion criteria were having been institutionalized for less than 3 months, suffering from a terminal disease, being blind and or deaf, exhibiting cognitive impairment with a score less than 18 on the Arabic Mini-Mental State Examination (AMMSE), and being below 60 years of age. Of the 2094 elderly people living in the 36 NHs, 354 residents (average age 76.84) were enrolled in the study. Only three elderly who were not excluded and did not respond were excluded from the study.

The authors sent a consent form to NH administrators describing the project and asking for approval to enter the NH and obtain information from the residents and the medical records. Administrators were told that all information would be anonymized. We garnered approval from 36 NH administrators which identified residents fitting the enrolment criteria. Field researchers described the project to potential participants, who were given a one-page summary of the project in Arabic and were informed that certain information would be taken from their medical records but that anonymity would be maintained. Residents who were willing to participate either signed a consent form or gave verbal consent in the presence of a nurse or caregiver. No duress was used when obtaining consent to participate or gathering responses to questions on the ADL.

Participants were first given the Arabic Mini Mental State Examination (AMMSE) to determine their cognitive abilities and to identify dementia. They then completed the Arabic version of the ADL. In some cases, caregivers answered the questions in the presence of the participants. In addition, data were obtained from the medical records, including disability measures and sociodemographic information. The field researchers maintained complete respect for the rights and needs of the participants.

Assessment data were coded by a data entry person. The codes were tagged to the caregiver and the NH data. The data were related through a coding scheme to maintain the anonymity of elderly, the NHs, and caregivers.

### The Mini-Mental State Examination

The MMSE was administered to assess the cognitive level of the participants. It has concurrent and construct validity, established by Folstein et al. [18], and was translated into Arabic by the same group [19]. It is a 30-item cognitive scale that tests orientation, attention, and immediate and short term recall through verbal instructions. It is scored from 0 to 30. A score less than 23 indicates possible cognitive impairment. However, for less educated Arab-speaking respondents, a lower cut-off, such as 20, can be used [20] and allow inclusion of the respondent in the study. Participants who received a score of less than 18 or who were diagnosed by a geriatrician as having normal cognitive function were excluded from the study.

## Results

When the three judges rated the three translations for adequacy, applicability, and appropriateness, there was an 83% agreement on the adaptability of the translated items and 100% agreement on the applicability and appropriateness. The items with weak adaptability were reviewed and translated by the judges then rated again. Once the judges reached 100% agreement on adaptability, they finalized the Arabic ADL instrument. The tool was tested in 10 elderly to see if the translations could be operationally defined, and this pilot study did not identify any problems.

Respondents were grouped according to age using the classification system of Vierck and Hodges [21]. The age group between 60 and 70 was classified as "young age", 75–84 as "middle age", and 85 and above as older old. The majority n = 159 of the participants was over 75 and 65% of the sample were female. A mean score was calculated for each ADL item (see Table 1) and for the total score. The large majority (see Table 2) of the sample did not have severe disabilities that would render them almost non-functional. A small number (8.3%) were severely dysfunctional. Almost 26% had cardiovascular disease, 3.3% had arthritis, and 17.6% had diabetes mellitus. Of the participants with diabetes, 87.1% had not had an amputation and 97.8% of those who had undergone amputation were not crippled, suggesting that these comorbidities had no direct relation with disability (Table 2).

**Table 1:** ADL Mean score on each of the items

	Mean (SD)
Bathing	0.55 (0.42)
Dressing	0.69 (0.42)
Going to the toilet	0.78 (0.37)
Transferring (movement)	0.83 (0.30)
Continence	0.84 (0.31)
Feeding	0.96 (0.16)
ADL-scale	0.77 (0.26)

**Table 2:** Demographic representation of the elderly sample

Age	Young old (60–74)	91 (36.4%)	
	Middle-old (75–84)	115 (46%)	

	Older-old (85-high age)	44 (17.6%)	

Gender	Male	87 (34.4%)	

	Female	166 (65.6%)	

ADL	Severe	21(8.3%)	

	Moderate	12(4.8%)	

	None to Mild	219(86.9%)	

cardiovascular disease	Yes	63	26.4%

	No	175	73.5%

High Blood Pressure	Yes	109	45.7%

	No	129	54.2%

Diabetes	Yes	42	17.6%

	No	196	82.3%

Arthiritis	Yes	8	3.3%

	No	230	96.6%

Disability			

Amputations	Yes	10 (4.0%)	

	No	239 (94.5%)	

Crippled	Yes	18 (7.1%)	

	No	228(90.1%)	

Other Disabilities	Yes	225 (88.9%)	

	No	20 (7.9%)	

Each of the variable attribute do not add to 100% because of missing responses

The reliability split half measure had a strong Cronbach alpha of 0.90 for the first three subscales on the ADL (bathing, dressing, and going to the toilet). The Cronbach alpha for the second three subscales was 0.65 for transferring (movement), continence, and feeding, with a correlation of r = 0.8 between the two halves.

Sensitivity, specificity, and positive and negative predictive values were calculated for each of the ADL items. A total score was computed for the ADL: a total score above 3 was considered negative (i.e. no dependence) and below or equal to 3 was considered positive (i.e. indicating dependence). The positive and negative predictive values were cross-referenced against the two conditions of being crippled or having had an amputation. A high probability for sensitivity and predictor value negative was found with low probabilities for specificity and predictive value positive.

The probability of the predictive value positive was very small compared to a high predictive value negative. Because of the high level of sensitivity of the ADL, there were few false negatives: the high negative predictive value therefore meant a high probability that elderly people who were not dependent screened negative on the ADL (see Table 3 and Table 4).

**Table 3:** Sensitivity and specificity measures among conditions

	Amputation Sensitivity	Amputation Specificity	Crippled Sensitivity	Crippled Specificity
Bathing	0.9	0.41	0.88	0.41
Dressing	0.6	0.62	0.83	0.65
Going to the toilet	0.7	0.73	0.83	0.75
Transferring (movement)	0.8	0.45	0.88	0.78
Continence	0.4	0.77	0.38	0.78
Feeding	0.1	0.94	0.05	0.94
ADL-scale	0.7	0.72	0.83	0.74

**Table 4:** Predictive value negative and predictive value positive for each condition

	Amputation Predictive value positive	Amputation Predictive value negative	Crippled Predictive value positive	Crippled Predictive value negative
Bathing	0.05	0.97	0.15	0.96
Dressing	0.06	0.97	0.15	0.95
Going to the toilet	0.10	0.97	0.21	0.94
Transferring (movement)	0.12	0.97	0.24	0.94
Continence	0.07	0.96	0.13	0.92
Feeding	0.06	0.95	0.10	0.91
ADL-scale	0.09	0.97	0.19	0.95

The relationship between age, as a risk factor for dependence, and the total score on the Arabic ADL was analyzed using the Pearson correlation. A low but negative correlation of r = -0.04 (p = 0.57) indicated that the higher the age group, the higher the ADL and, therefore; the greater the functional dependence.

To determine the validity of using an AMMSE score cut-off of 18 for cognitive impairment, we compared the total ADL score of the group with AMMSE 18–20 to a second group scoring above 20, using Student's t-test (respondents scoring below 18 were excluded from the study). The mean ADL score for the lower group (AMMSE 18–20) was 71.2 (SD = 35.1). It was higher for the higher AMMSE group at 78.69 (SD = 25.69) but this difference was not significant t(df = 250) = 1.13, p = 0.26.

We looked at whether the ADL could discriminate between the older old, middle-old and young-old. No significant differences were found, F(2,246) = 0.14, p = 0.86. However, the older old had lower ADL scores than the middle and younger age groups, at a non-significant level (Table 5).

**Table 5:** Means and SD for Age and AMMSE groups on the percentage ADL score

	Mean	SD
Age		
		
Young old (60–74)	78.02	26.92
Middle-old (75–84)	78.36	27.31
Older-old (85-high age)	75.87	24.15
		
AMMSE		
		
18–20	71.2	35.1
21–31	78.69	25.69

## Discussion and conclusion

Many caregivers for the elderly use general observation and clinical judgment as easy, practical and quick methods for assessing the activities of daily living. Having an objective, reliable and valid instrument for Arab speaking clients would be useful in the Mediterranean and Middle East. In our review of the literature, not one study used an Arabic, valid and reliable ADL instrument.

We used the ADL, a reliable, valid instrument that has been field tested in Lebanon. We translated the ADL and assessed whether it could provide objective indications of dependence. The translation was improved by a committee of three experts who rated the items on adaptability, applicability, and appropriateness. Agreement by the judges in the first round of translation reached 94.3% on these three factors. Through a process of reiterative translation and rating, judgment reached 100% agreement.

The translated ADL was administered to elderly people living in nursing homes. The high Cronbach alpha of the ADL, indicated 3 random items correlated highly with the rest of the three items.

Ideally, for a test to be a maximally useful screening tool, both sensitivity and specificity should be 100%, so that every subject with the condition has a positive test (no false negatives), and there is a negative predictive value of 100%. Similarly, positive tests should occur only in subjects with the condition (no false positives), resulting in a positive predictive value of 100%, which is generally impossible [17]. Considering the intended purpose and use of the ADL, it was logical to use sensitivity to see whether a negative result ruled out that the screened person would be dependent. It may seem obvious that people who screen positive for dysfunctional conditions have more noticeable problems than those who are at the borderline. Thus, a sensitive screening instrument and a negative predictive value with a high probability of those screened with a negative result of the condition, do not have the condition provides evidence of confirmation. As expected, the Arabic ADL had a high sensitivity and high negative predictive value.

The negative predictive value gives confidence in the use of the chosen scoring scheme, involving scores of 0, 0.5 or 1. The 0.5 was transformed to a 0 as a positive measure of the condition and this would increase the risk of false positives. With low proportions in the sample having either of the two test conditions we chose as likely to increase dependence (being crippled, having had an amputation), an appropriate test would have a high sensitivity and a high negative predictive value, minimizing falsely negatives.

Possibly because the elderly in our sample were generally independent, as shown in the mean overall ADL score (Table 1), the feeding subscale was a poor predictor of dependence. Neither being a cripple nor having had an amputation altered the sensitivity of the feeding subscale. On the other hand, as reported by Gill et al. [22], it may be that caregivers in NHs make special efforts to feed all elderly patients, irrespective of their physical condition, for example, trying to ensure that patients consume similar food portions, even if they can feed themselves. To test this proposition, future studies could compare feeding as a predictive validity measure in people living in NHs and among independent free-living older people.

The literature indicates that the development of dementia is associated with disability [23]. The ADL has been shown to be sensitive to functional changes in individuals with mild to moderate dementia [24,25]. We assessed whether using a cut-off score of 18 on the AMMSE to indicate cognitive impairment was related to dependence or independence on the ADL. If differences existed, this would suggest the cut-off was reasonable. There was no difference between the subjects scoring 18–20 on the AMMSE compared to those scoring above 20 in terms of their overall ADL score; however, a higher mean on the ADL was found in those who scored above 20 on the AMMSE.

We investigated whether increasing age, a known a risk factor for loss of independence, predicted a lower overall ADL score and found no association. In fact, with 8.3% of the sample being severely handicapped, dependence would be associated with the age rather than condition. However, the negative correlation found between age and the ADL percentage score is substantiated by Covinsky, et al. [26] that suggested the older old were more likely to have most other risk factors found through the set conditions.

Assessing the activities of daily living is essential for planning geriatric interventions [26]. For example, exercise can have positive health outcomes for people who start exercising at a later age [27,28]. A quick, easy assessment instrument in Arabic would support caregivers and geriatricians in assessing elderly patients in Lebanese NHs and prescribing appropriate physical exercise. The Arabic ADL appears to be such an instrument.

## Competing interests

The authors declare that they have no competing interests.

## Authors' contributions

RN, wrote and conceptualized the study presented in this paper. JD administered the questionnaires collected the data, conceptualized and edited the paper for the study. All authors read and approved the final manuscript.

## Pre-publication history

The pre-publication history for this paper can be accessed here:



## Supplementary Material

Additional file 1 **Arabic ADL Scale**. The Arabic ADL scale is presented with a 0, 1/2 and 1 response format. Click here for file
