# Supplementary figures and images for: Validity and reliability of the Arabic version of Activities of Daily Living (ADL)
Source: BMC Geriatr. 2009 Mar 29;9:11. doi: 10.1186/1471-2318-9-11 (PMC2670307; doi:10.1186/1471-2318-9-11)

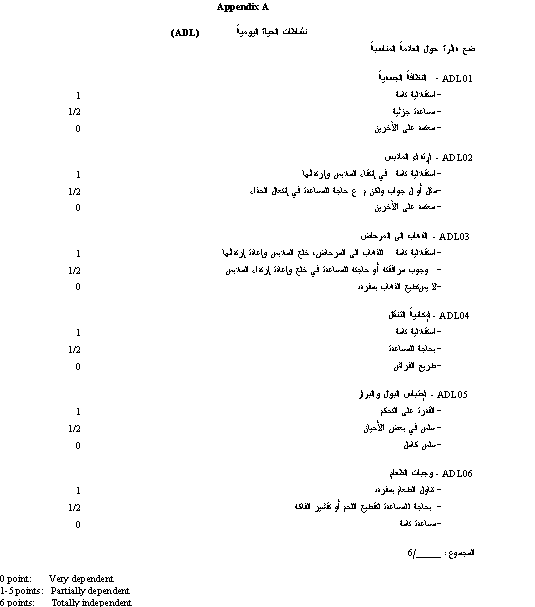

Supplement: Additional file 1 — Arabic ADL Scale. The Arabic ADL scale is presented with a 0, 1/2 and 1 response format. [file 1471-2318-9-11-S1.bmp]
